# Importance of Hyperbilirubinemia in Differentiation of Primary and Secondary Hemophagocytic Lymphohistiocytosis in Pediatric Cases

**DOI:** 10.4084/MJHID.2014.067

**Published:** 2014-11-01

**Authors:** Seval Ozen, Alper Dai, Enes Coskun, Serdar Oztuzcu, Sercan Ergun, Elif Aktekin, Sibel Yavuz, Ali Bay

**Affiliations:** 1 Gaziantep University Department of Pediatrics, Gaziantep, Turkey; 2 Gaziantep University Department of Medical Biology, Gaziantep, Turkey; 3 Gaziantep University Division of Pediatric Hematology Gaziantep, Turkey

## Abstract

**Background and objective:**

Hemophagocytic lymphohistiocytosis (HLH) is a life-threatening hyper-inflammatory disease. It is difficult to differentiate between primary and secondary HLH based on clinical findings at the onset of disease. We aimed to find parameters that can help to differentiate primary and secondary HLH at initial diagnosis especially for physicians working in developing countries.

**Patient and Method:**

We retrospectively analyzed data of 38 HLH patients who were admitted to the Pediatric Hematology Department of Gaziantep University between January 2009 and December 2013.

**Results:**

Of 38 patients, 20 were defined as primary, and 18 were secondary HLH. The average age of primary and secondary HLH patients was 31±9 and 81±14 months, respectively (p=0.03). We found consanguinity rates significantly higher in primary HLH patients compared to secondary HLH patients (p=0.03). We found that total and direct bilirubin levels significantly increased in primary HLH patients compared to secondary HLH patients (p=0.006, p=0.044). Also, CRP levels were found markedly increased in secondary HLH patients compared to primary ones (p=0.017).

**Conclusion:**

We showed that cholestasis and hyperbilirubinemia findings of HLH patients at the initial diagnosis should be considered in favor of primary HLH, and an increased level of CRP should be considered in favor of secondary HLH.

## Introduction

Hemophagocytic lymphohistiocytosis (HLH) is a life-threatening hyperinflammatory disease caused by an uncontrolled and dysfunctional immune response.^1^ HLH has been categorized as primary or familial HLH (FHLH), when there is a family history of HLH or known underlying genetic defects. Reactive or secondary HLH occurs in the setting of infection or underlying rheumatologic disorders or malignancy.^2^ HLH occurring in the setting of a rheumatological illness is commonly referred to as macrophage activation syndrome (MAS).

However, initial treatment should not govern disease classification (genetic or acquired). However, information about the underlying genetic defect is important for management because it will allow for an early search for a stem cell donor.^3^ Differentiation between primary and secondary forms of HLH has become increasingly blurred together as new genetic causes are identified.^4^ In many developing countries, these genetic tests are not performed, and blood had to be sent abroad for genetic testing. Elongation of the process causes difficulties in the follow-ups of these patients. It is difficult to differentiate between primary and secondary HLH based on clinical symptoms, history of infection, or the early clinical course at the onset of disease.^5^ Severity of disease and the identification of an infectious agent do not differentiate between genetic and acquired forms of HLH. Age is helpful to some extent a minority of children one year of age will have acquired HLH, but older age does not reliably exclude genetic HLH.^6^

In this study, we aimed to find parameters that can help to differentiate primary and secondary HLH at initial diagnosis by comparing clinical laboratory findings of 38 HLH patients followed in our clinic for last four years. This procedure can be particularly useful for physicians working in developing countries.

## Patients and Methods

From January 2009 to December 2013 we diagnosed 38 patients as HLH according to Diagnostic Guidelines for HLH 2004.^7^

Patients who were found to have a genetic abnormality and/or early-onset disease (≤2 yr) with family history were considered as having familial HLH. Patients whose genetic testing for UNC13D, PRF1, STX11, and STXBP2 revealed no genetic abnormality and who had no family history of HLH were considered as having secondary HLH.

20 of 38 patients with genetic mutations detected or who had at least one of the following conditions; family history or parental consanguinity, persistence or recurrence of HLH, were classified as having primary HLH. The remainder 18 patients without a genetic mutation detected and who unmet the conditions mentioned above were classified as secondary HLH.

All patients fulfilled at least five fundamental criteria of HLH at the time of diagnosis, including fever, hepatosplenomegaly, bicytopenia and/or pancytopenia, hypertriglyceridemia and/or hypofibrinogenemia, hyperferritinemia, and hemophagocytosis in the bone marrow. Patients were evaluated regarding with age, clinical findings, and laboratory data using by descriptive statistics.

## Statistical methods

SPSS.20 statistical software was used for the analysis. Student’s t test and Mann–Whitney U test were used, and p value less than 0.05 was evaluated as statistically important.

## Results

A total of 38 HLH patients is included into this study. Of 38 patients, 20 were defined as primary, and 18 were secondary HLH. Perforin, sytaxin, and munc13-4 mutations were detected in 6, 3, and 1 of primary HLH patients, respectively. The remaining ten patients were considered to have primary HLH based on family history, age of onset and recurrence of the disease, even if the genetic mutations were not detected.

Out of 20 patients with primary HLH, 12 (60%) were female, and 8 (40%) were male. Out of 18 patients with secondary HLH, 11 (61%) were female and 7 (39%) were male (39%). The average age of primary and secondary HLH patients was 31±9 and 81±14 months, respectively. Patients with primary HLH were significantly younger than those with secondary HLH (p=0.03) (Table 1).

We found consanguinity rates significantly higher in primary HLH patients compared to secondary HLH patients (p=0.03). Also, the sibling death history was present in 30% of primary patients but none in secondary HLH patients. We did not detect any significant difference between primary and secondary HLH patients when compared their clinical findings such as fever, hepatomegaly, and splenomegaly. Also, distribution of ethnicity can be seen on Table 1.

We also compared the laboratory findings of HLH patients and; we found that total and direct bilirubin levels significantly increased in primary HLH patients compared to secondary HLH patients (p=0.006, p=0.044) (Table 2). When we took the cut-off level 1,3 mg/dl for total bilirubin level, we calculated the sensitivity and specificity levels 60%, 95% respectively. By the way when we took the cut-off level 0,8 mg/dl for direct bilirubin levels, sensitivity and specificity levels were calculated 60% and 95% respectively (Table 3). Also, CRP levels were found markedly increased in secondary HLH patients compared to primary ones (p=0.017). We calculated the sensitivity and specificity levels 47% and 85% respectively, when we took the cut-off level 98 mg/dl (Table 3). We didn’t find any significant difference between the two groups by comparing the levels of WBC, hemoglobin, platelet, triglyceride, fibrinogen, ferritin, transaminase, albumin, LDH, Na, K, P, Ca, PT, INR, aPTT, and the sedimentation rate.

## Discussion

The exact incidence or prevalence of HLH is not known. Based on the available data, the incidence of HLH varies by geographic region.^8^ It has been reported to occur in anywhere from 1 of 50 000 live births in Sweden^9^ to 7.5 of 10 000 live births in Turkey;^10^ this unusually high reported prevalence is attributed to increased consanguinity. Familial HLH comprises about 25% of all HLH, a number that is more likely going to increase in coming years with the recent boom in sequencing and genetic testing.^11^,^12^ Acquired HLH, which makes up majority of HLH in both children and adults, is not associated with a known genetic defect by definition. The hyper-inflammatory state is triggered by infectious, autoimmune, or neoplastic conditions.^13^

The diagnostic criteria for HLH have been developed and updated in 2004 by the FHL Study Group of the Histiocyte Society.^7^ Making the diagnosis of HLH is not sufficient. Identifying whether the patient has genetic or acquired disease is also important for the management of the disease. The treatment can be stopped in secondary HLH patients after controlling the acute episode.^14^ But in genetic, persistent and recurrent HLH, continuation of treatment is recommended until SCT (stem cell transplantation) is done.^15^ Rapid detection of genetic disease can provide an opportunity to start searching for an appropriate donor quickly; also give the chance of starting an immediate treatment and follow-up until a donor is available.^16^

It is important to predict whether patients have primary or secondary HLH at the first admission in especially developing countries since the investigation of HLA groups for stem cell transportation takes a long time. In the literature, certain criteria for differentiation between primary and secondary HLH have not been specified yet.^8^,^13^ Impaired NK cell cytotoxicity is a characteristic finding in FHL and immunodeficiency syndromes with albinism; however, normal activity does not exclude either.^6^,^17^ Moreover, a decreased function of NK cells has been observed in patients with acquired HLH, in patients with MAS, and in close relatives of patients with FHL.^18^

Recently, flow cytometry has been used as a screening method to identify patients with a genetic predisposition to HLH.^6^,^19^ Intracellular stains detecting perforin, SAP (X-linked lymphoproliferative syndrome [XLP-1]), and XIAP (XLP-2) are available. Munc13-4 protein expression in platelets has been reported as potential new rapid screen for FHL-3 and was awaiting testing in a larger cohort.^20^ Genetic defects with impaired granule exocytosis (FHL 3–5, CHS, and GS-2) lead to impaired translocation of the lysosome-associated membrane glycoprotein CD107a to the cell surface, upon stimulation of NK cells or cytotoxic T lymphocytes (CTLs).

In 494 patients evaluated within a collaborative European study, the NK degranulation assay clearly differentiated between patients with defects in granule exocytosis and patients with acquired HLH or other hereditary defects, such as perforin, SAP, or XIAP deficiency.^21^ Once these functional tests suggest a genetic basis for HLH, molecular analysis should be followed, including for parents and siblings.

Although some articles suggesting that NK cell degranulation could be used for differentiation of primary and secondary HLH have been published but these methods are very hard to be used in developing countries. These tests also require financial strength and special dyes in flow-cytometry together with a certain experience. We investigated whether differentiation of primary and secondary HLH is possible just with clinical findings and laboratory tests which can be applied in every facility easily. In our study, consanguinity and history of sibling death rates were found significantly higher in primary HLH patients as consistent with the literature when we compared the primary and secondary HLH patients. The most intriguing finding in our study was that total and direct bilirubin levels were increased in a manner statistically significant in primary HLH patients compared to secondary ones. This finding has not been reported in the literature before. Recent study published by Japan Histiocytosis Study Group evaluated prognostic factors of Epstein–Barr virus-associated hemophagocytic lymphohistiocytosis in children.^22^ They reported significantly higher total bilirubin levels in non-survivors than in survivors. More recently, it was indicated in a study reported from Vietnam that hyperbilirubinemia on admission will be useful and could be a practical predictor to determine high-risk HLH patients.^23^ However, there was no differentiation of primary and secondary HLH in those studies.

Our second finding is CRP levels found markedly increased in secondary HLH patients compared to primary ones (p:0,017). In a study reported by Stephan et al.,^24^ CRP levels greater than 50 mg/L have been associated with increased risk of infection and overall mortality in HLH patients with underlying autoimmune disorders. Thus, ESR or CRP may be used as indices of disease severity, but care must be taken to identify coincident inflammatory insults such as infection or autoimmune disease. There are no patients with complicating bacterial infection in our secondary HLH group. There are little data showing increased level of CRP in HLH patients. In studies conducted on adult patients mostly, CRP levels were detected as higher in HLH group when compared to not having HLH. In pediatric and adult age groups, there were no studies comparing CRP levels between the primary and secondary HLH. A possible cause of CRP increase is that stimulants needed for the development of HLH should be stronger due to the absence of a genetic defect in secondary HLH patients. Therefore, this may be speculated as like that CRP level is higher during more severe inflammation developing as a result of strong stimulants. Therefore, this situation may explain the increase of CRP in secondary HLH.

In our study, we investigated the criteria that can be used in the differentiation of FHL and secondary HLH for the patients especially in countries not having advanced laboratory facilities. As a result, we showed that the cholestasis and hyperbilirubinemia, found in HLH patients at the initial diagnosis, should be considered in favor of primary HLH. On the contrary, an increased level of CRP should be considered in favor of secondary HLH. These data should be confirmed being our study conducted for a limited number of patients in a single center.

## Figures and Tables

**Table 1:** Patient Characteristics, Age and Gender Distributions

		Primary (n=20)	Secondary (n=18)	P

**Age (months)**		31±9	81±14	**p=0.03**

**Female/male**		12/8	11/7	p>0.05

**Consanguinity**	**Present**	18 (90%)	8 (44.4%)	**p=0.03**
		
	**Absent**	2 (10%)	10 (31.6%)	

**History of Sibling Death**	**Present**	6 (30%)	0	**p=0.021**
		
	**Absent**	14 (70%)	18 (100%)	

**Fever**		19(95%)	18(100%)	p>0.05

**Splenomegaly**		20(100%)	17(94.4%)	p>0.05

**Hepatomegaly**		19(95%)	15(83.3%)	p>0.05

**Turkish**		12(60%)	15(83.3%)	

**Arabic**		4(20%)	2(11.1%)	

**Kurdish**		4(20%)	1(5.6%)	

**Table 2:** Biochemical Parameters of the Patients

	Primary HLH	Secondary HLH	p value
	Mean (Min-Max)	Mean (Min-Max)	
**AST (5–45 U/L)**	396 ± 200 (48–4138)	272 ± 87 (15–1300)	p>0.05
**ALT (5–45U/L)**	286 ± 202 (1–4120)	152 ± 61 (13–1082)	p>0.05
**Total bilirubin (<2mg/dl)**	3 ± 0.6 (0.3–7.5)	0.6 ± 0.09 (0.2–1.7)	**p=0.006**
**Direct bilirubin (<0.2 mg/dl)**	2.3 ± 0.5 (0–6.4)	0.4 ± 0.08 (0.1–1.6)	**p=0.044**
**Albumin (3.5–5.2 g/dl)**	2.7 ± 1.1 (1.4–3.7)	3 ± 0.14 (2–4.5)	p>0.05
**LDH (100–295 U/L)**	793 ±169 (110–2895)	1503 ± 468 (321–7190)	p>0.05
**Na (133–146 mmol/L)**	128 ± 1.1 (116–136)	130 ± 0.8 (125–138)	p>0.05
**K (3.4–5.1 mmol/L)**	4.2 ± 0.1 (3.5–5)	4 ± 0.1 (2.6–5)	p>0.05
**P (4.5–5.5 mg/dl)**	3.5 ± 0.28 (2–7.5)	3.6 ± 0.4 (1.7–7.8)	p>0.05
**Ca (8.8–10.8 mg/dl)**	8.1 ± 0.3 (7–9.5)	8 ± 0.2 (5.9–9.6)	p>0.05
**Sedimentation rate (4–20 mm/hour)**	23 ± 5 (2–64)	39.7 ± 7.9 (1–115)	p>0.05
**C-reactive protein (0–0.5 mg/dl)**	46 ± 10 (4–185)	104 ± 16.9 (3–197)	**p=0.017**
**Triglyceride (mg/dl)**	397±47 (120–936)	364±76 (47–1350)	p>0.05
**Ferritin (ng/ml)**	11500 ±3282 (405–56124)	11200±2895 (1574–40000)	p>0.05

**Table 3:** Sensitivity and Specificity Analysis of Total Bilirubin, Direct Bilirubin, and C-reactive protein

Variable	Sensitivity (%)	Specificity (%)	Cut off
Total Bilirubin	60	95	1,3 mg/dl
Direct Bilirubin	60	95	0,85
C- Reactive Protein	47	85	98
